# Degradation of Proteins From Colostrum and Mature Milk From Chinese Mothers Using an *in vitro* Infant Digestion Model

**DOI:** 10.3389/fnut.2020.00162

**Published:** 2020-09-16

**Authors:** Mohèb Elwakiel, Sjef Boeren, Wendan Wang, Henk A. Schols, Kasper A. Hettinga

**Affiliations:** ^1^Food Quality and Design Group, Wageningen University & Research, Wageningen, Netherlands; ^2^Laboratory of Food Chemistry, Wageningen University & Research, Wageningen, Netherlands; ^3^Laboratory of Biochemistry, Wageningen University & Research, Wageningen, Netherlands; ^4^Inner Mongolia Yili Industrial Group Co., Ltd., Hohhot, China

**Keywords:** human milk proteins, protease inhibitors, casein, serum proteins, milk protein digestion

## Abstract

This study provided insights into the degradation of human milk proteins in an *in vitro* infant digestion model by comparing colostrum (week 1) and mature milk (week 4) of 7 Chinese mothers individually. In this study, we adapted the exiting INFOGEST *in vitro* model, to conditions representative to infants (0 to 3 month-old). The level of undigested proteins was analyzed by LC-MS/MS after gel-electrophoretic separation and in-gel digestion. The BCA protein assay showed that the total undigested milk protein content decreased from the start to the end of digestion with variations between mothers, especially in the gastric phase (25–80%). Undigested proteins could also still be found after the intestinal phase, ranging from 0.5 to 4.2% of initial protein content. Based on LC-MS/MS analysis, milk protein digestion varied between the mothers individually, especially during the gastric phase. No differences could be observed between protein digestion from colostrum and mature milk after the intestinal phase. The highest levels of proteins remaining after intestinal digestion can be linked to the group immune-active proteins, for all mothers. The level of protease inhibitors and total protein content in the milk did not correlate with the overall proteolysis during digestion. The results also showed that milk serum proteins partly remained after the gastric phase, with 33% remaining from colostrum and 37% remaining from mature milk. More than 40 milk serum proteins were detected after the intestinal phase. Some of the highly abundant milk serum proteins (lactoferrin, serum albumin, bile salt-activated lipase, immunoglobulins, α_1_-antichymotrypsin) were still partially present intact after the intestinal phase, for all mothers. Caseins were also not completely digested in the gastric phase, with 35% remaining from colostrum and 13% remaining from mature milk. Caseins, on the other hand, were almost completely digested after the intestinal phase. The complete degradation of caseins into peptides might be related to their structural features. Overall, this study showed that digestion differed for the various human milk proteins by adapting an *in vitro* digestion model to infant physiological conditions, with the main differences between digestion of the milk from individual mothers being observed after gastric digestion.

## Introduction

Human milk is a complex mixture of nutrients and bioactive constituents, contributing to the infant's growth, development and health (1). Human milk proteins, amongst others, play a pivotal role in protecting the infant's gut mucosa against pathogens (1). There are two distinct types of proteins in human milk, caseins, and serum proteins, with changing quantities and ratios over lactation (1). Caseins (α_S1_, β-, and κ-casein) are generally described as transport proteins due to their calcium-binding properties, and become bioavailable after digestion (2). The hydrophobic regions of caseins consist of a high number of proline residues, which prevents the formation of close-packed secondary structures (3). The serum proteins in human milk have many different functions (4). The most abundant serum protein groups in human milk are enzymes (e.g., α-lactalbumin, bile salt-activated lipase), transport proteins (e.g., serum albumin, fatty acid-binding protein), and immune-active proteins (e.g., lactoferrin, immunoglobulins) (5). This latter study also showed that the milk serum protein composition varied among mothers and between different populations (5).

It has been assumed that caseins in human milk are fully digested in the infant's digestive tract, facilitating the uptake of relatively small peptides, essential amino acids and minerals associated with the micelles (6–8). Human milk serum proteins that do not have an extensive tertiary structure, e.g., polymeric immunoglobulin receptor (PIGR) and osteopontin, also may be broken down completely during infant digestion, in contrast to more tightly folded milk serum proteins like lactoferrin and immunoglobulins (9–13). Some of the major proteins (caseins, osteopontin, clusterin, PIGR) may be predigested by proteases, resulting in the presence of peptides in undigested human milk (12). A variety of studies have reported that specific serum proteins like lactoferrin, immunoglobulins, and α_1_-antitrypsin from human milk can be found intact in the stool of breastfed infants, showing that those proteins are able to partially survive digestion in the infant's digestive tract (14–18).

It has been suggested that the extent of protein digestion might be reduced by the presence of protease inhibitors (e.g., α_1_-antichymotrypsin and α_1_-antitrypsin) or by the high total protein levels in human milk. Protease inhibitors might inhibit the function of trypsin and other serine proteases during small intestinal digestion (19–22). Colostrum contains a relatively higher quantity of protease inhibitors than mature milk (5), which might lead to more undigested proteins from colostrum at the end of digestion. In addition, protein digestion might also be influenced by the total protein content in colostrum. Colostrum contains a higher total protein content (14–16 g/L) compared to mature milk (7–10 g/L) (1). Therefore, it would be of interest to investigate both the variation in level of protease inhibitors, as well as total protein content, in relation to the degree of protein hydrolysis.

Different static *in vitro* digestion models have been developed over the years, mimicking the gastrointestinal tract of adults and 3 year-old infants (23–31). Although static *in vitro* digestion only partly represents the digestive tract, the INFOGEST adult *in vitro* model has been shown to mimic *in vivo* digestion of bovine milk proteins (32, 33). Colostrum (between 0 and 2 weeks) and mature milk (>4 weeks postpartum) are quite different in protein content and composition (4). Based on the gene expression of the infant's gastrointestinal tract, infants at 4 weeks of age have amongst others 40% of their chymotrypsin capacity, and only 10% of their pepsin capacity available compared to adults (34). To elaborate and better comprehend the digestion of proteins from colostrum and mature milk, the preexisting INFOGEST *in vitro* digestion model was adapted representing 0 to 3 month-old infant's digestion. This model is different from the existing adult *in vitro* digestion model (23), by having a higher gastric pH of 5, lower enzyme activities (pepsin 200 U/mL in gastric phase; trypsin 8.33 U/mL in intestinal phase), and shorter transition times (1 h each for gastric and intestinal phases) (34). Based on these modifications, this would further mimic the situation in infants, who have generally decreased protein digestion than adults.

The aim of this study was to better understand the variation in protein digestion. The enzymatic hydrolysis of proteins in milk from 7 Chinese mothers from 2 different lactation periods (colostrum, week 1; mature milk, week 4) was investigated in an adapted *in vitro* digestion model representing 0 to 3 month-old infants. The level of undigested proteins was analyzed by a combination of bicinchoninic acid (BCA) protein assay, sodium dodecyl sulfate polyacrylamide gel electrophoresis (SDS-PAGE) and liquid chromatography–tandem mass spectrometry (LC–MS/MS).

## Materials and Methods

### Sample Collection

Human milk was collected as described previously (5) and samples from 7 different Chinese mothers from 2 different stages of lactation (colostrum, week 1; mature milk, week 4) were used. The samples used in this study were aliquots, but not the same samples, as described previously (5). Healthy Chinese women who delivered singleton term infants (38–42 weeks) were eligible for this study. Human milk collection was approved by the Chinese Ethics Committee of Registering Clinical Trials (ChiECRCT-20150017). Written informed consent was obtained from these 7 mothers.

### The Infant *in vitro* Protein Digestion Model

The INFOGEST static adult *in vitro* digestion model, as described previously (23), was modified to an *in vitro* infant (0–3 months) protein digestion model (34). In comparison to adults, the pH and porcine pepsin concentrations in the gastric phase were adjusted, as well as the porcine pancreatin and porcine bile salt concentrations in the intestinal phase. The time to mimic each digestion phase was changed to 1 h. Briefly, the milk fat was removed by centrifugation (10 min, 1,500 g, 4°C). Next, skim milk (8 mL) was mixed with 6 mL of simulated gastric fluid, after which 5 μL of 0.3 M calcium chloride (CaCl_2_) and 695 μL of water was added. Porcine pepsin diluted in simulated gastric fluid was added to reach an enzyme activity of 200 U/mL instead of 2,000 U/mL for the adult model in the final gastric mixture. The pH of the chyme was adjusted to 5 instead of 3 with 1 M HCl. The mixture was then incubated at 37°C for 1 h while mildly shaking at 200 rpm. After incubation, the pH was adjusted to 7 with 1 M sodium hydroxide solution. For duodenal digestion, 7.5 mL of gastric chyme was mixed with 4 mL of simulated intestinal fluid electrolyte stock solution (23). Porcine pancreatin was added to reach a trypsin enzyme activity of 8.33 U/mL instead of 100 U/mL for the adult model. After that, 2.5 mL of bile salts (40 mM), 40 μL of 0.3 M CaCl_2_, and 1.31 mL water was added. The pH of the chyme was then again adjusted to 7 with 1 M hydrogen chloride solution. The duodenal chyme was then incubated at 37°C for 1 h in a water bath while mildly shaking at 200 rpm. After incubation, the porcine pancreatin was inactivated with 50 μL of the irreversible serine protease inhibitor 4-(2-aminoethyl) benzenesulfonylfluoride (100 mM) in the duodenal chyme. The skim milk was diluted 4 times, while the samples after the gastric phase were diluted twice, facilitating direct comparison with the samples of the duodenal phase (34).

### Total Concentrations of Proteins Before and After *in vitro* Digestion

The total protein concentration of the blank and digesta were measured in duplicate using the BCA protein assay kit 23225 (Thermo Scientific Pierce, Massachusetts, U.S.). Standards and reagents were prepared according to the manufacturer's instructions. Before analysis, 1 mL of the sample was mixed 1:1 with absolute trichloroacetic acid (TCA). After centrifugation (1,500 g for 30 min, 4°C), the supernatant containing peptides was removed. TCA-precipitated proteins were washed twice with cold acetone to completely remove TCA, and the pellet dried at 70°C in a heating block (Labtherm Graphit, Liebisch, DE) for 60 min. The dried proteins were re-dissolved in 2 mL of the BCA working reagent, and incubated at 37°C in a water bath for 30 min. After cooling down to room temperature, the samples were ready for spectrophotometric measurements. Based on the BCA calibration curve using bovine serum albumin, concentrations were expressed as g/L for the diluted samples.

### SDS-PAGE, In-gel Digestion, and Purification by Solid Phase Extraction

For every sample, 2 μL was taken and diluted in 5 μL lithium dodecyl sulfate (LDS) sample buffer (pH 8.4, Life Technologies, Carlsbad, U.S.) and 15 μL water. This mixture was centrifuged at 1,500 g for 1 min and the supernatant heated at 70°C in a heating block for 10 min. Samples and pre-stained marker (5–165 kDa, Jena Bioscience, De) were then loaded onto NuPAGE 12% Bis-Tris gels (Life Technologies, Carlsbad, U.S.). Gels were run with an LDS running buffer (containing 50 mM MES, 50 mM Tris base, 0.1% SDS, 1 mM EDTA, pH 7.3) under non-reducing conditions at 120 V in a vertical electrophoresis cell (Bio-Rad, Hercules, U.S.). The gels were stained with colloidal Coomassie G-250 (Bio-Rad, Hercules, CA), followed by destaining with water and washing buffer (10% ethanol, 7.5% acetic acid in water). The gels were scanned after staining with Image Lab version 4.1 (Bio-Rad) to visualize the protein patterns and determine the location of α-lactalbumin on the gel.

In-gel digestion was used to digest proteins into peptides. As described previously (35), SDS-PAGE gels were incubated in 25 mL of ammonium bicarbonate (ABC) containing 0.039 g dithiothreitol (= 10 mM DTT, pH 8) for 1 h at 60°C. Subsequently, the gels were incubated in 25 mL of Tris buffer pH 8 containing 0.092 g iodoacetamide (= 20 mM IAA, pH 8) for 1 h at room temperature in the dark. To separate proteins (> 10 kDa) from small peptides, single lanes of the SDS-PAGE gels were cut into <1 mm^3^ small pieces and pieces above the band of α-lactalbumin were transferred to 1.5 mL Eppendorf low protein binding tubes. The gel pieces were frozen and thawed 3 times to increase the accessibility for trypsin. Then, 100 μL of 50 mM ABC (pH 8) containing 0.5 μg trypsin was added to the gel pieces, followed by 100 μL ABC to cover the gel pieces completely. According to the manufactures, the activity of bovine sequencing grade trypsin was ≥7,500 benzoyl-L-arginine-ethyl-ester U/mL protein (Roche, Basel, CH). After trypsin digestion overnight, 15 μL of 10% trifluoroacetic acid in water was added to adjust the pH between 2 and 4 (pH-indicator strips).

Solid phase extraction was done to purify peptides, as described previously (12). Stage tips containing 2 mg Lichroprep C18 (25 um particles) column material (C18+ Stage tip) were made in-house. The peptides were transferred to a methanol washed and 0.1% formic acid equilibrated C18 stage tip column. The peptides were eluted with 50 μL of 50% acetonitrile in water containing 0.1% formic acid. The samples were dried in a vacuum concentrator (Eppendorf, Nijmegen, NL) at 45°C for 1 h until the volume of each sample decreased to 15 μL or less. The content of the tubes was transferred to empty low protein binding tubes, and samples reconstituted to 50 μL by adding water containing 0.1% formic acid.

### LC-MS/MS and Data Analysis

LC-MS/MS was used to measure the amounts of distinct peptides. As described previously (35), 18 μL of each sample was injected on a 0.10 × 30 mm ProntoSil 300-3-C18H (Bischoff, Leonberg, DE) pre-concentration column (prepared in house at a maximum of 270 bar), peptides were eluted from the pre-concentration column onto a 0.10 × 200 mm ProntoSil 300-5-C18H analytical column, and the full scan FTMS spectra were measured in positive mode between m/z 380 and 1,400 on a LTQ-Orbitrap XL (Thermo Fisher Scientific, Waltham, U.S.). MS/MS scans of the four most abundant doubly- and triply-charged CID fragmented peaks in the FTMS scan were obtained in data-dependent mode in the linear trap (MS/MS threshold = 5.000) (35).

MS/MS spectra for each run were obtained and analyzed using the built-in Andromeda search engine with the Uniprot human protein database (36). Protein identification and quantification was done as described previously (4). Maxquant created a decoy database consisting of reversed sequences to calculate the false discovery rate (FDR). The FDR was set to 0.01 on peptide and protein level. The minimum required peptide length was seven amino acids, and proteins were identified based on a minimum of two distinct peptides. The intensity based absolute quantification (iBAQ) values were used, representing the total peak intensity as determined by Maxquant for each protein, after correction for the number of measurable peptides (5). The iBAQ values have been reported to have a good correlation with known absolute protein amounts over at least four orders of magnitude (37).

The Uniprot database was also used to assign functions to all individual identified proteins, as described previously (5). The iBAQ values for each protein were used individually and summed per function, and per digestion phase (predigestion, gastric phase, intestinal phase). The iBAQ values of the proteins individually and grouped per function per phase were also converted in percentages of the total iBAQ intensity. The total iBAQ intensities of the skim milk from colostrum and mature milk were set to 100%.

To compare colostrum and mature milk from 7 Chinese mothers on total protein (based on the BCA protein assay) after both gastric and intestinal digestion, a *t*-test for independent samples was used (R, Lucent Technologies, New York, U.S.). For this comparison, the total BCA protein concentrations were preferred over the summed iBAQ values. For statistical analysis, an FDR adjusted *p* < 0.05 was considered significant. Scatterplots were generated with both the total protein (based on the BCA protein assay) and the levels of protease inhibitors (iBAQ values) in human milk against the relative change of total protein from milk to gastric and to intestinal digestion.

## Results and Discussion

### Determination of Total Protein Before and After Digestion by the BCA Protein Assay

The existing INFOGEST *in vitro* digestion model was adapted to represent 0 to 3 month-old infant's digestion. The parameters of this adapted *in vitro* model, which was based on literature (34) including references and citations in that paper, represents the *in vivo* infant digestive conditions better than the adult model. Bovine milk serum was used for method development and validation of the model, as more was known about *in vitro* digestion of bovine milk proteins in older infants (2, 6, 11). As the findings for bovine milk were showing similar trends with previous studies (2, 6, 11), although using different age-specific models, the 0 to 3 month-old infant *in vitro* digestion model was then used for human milk samples.

**Figure 1:**
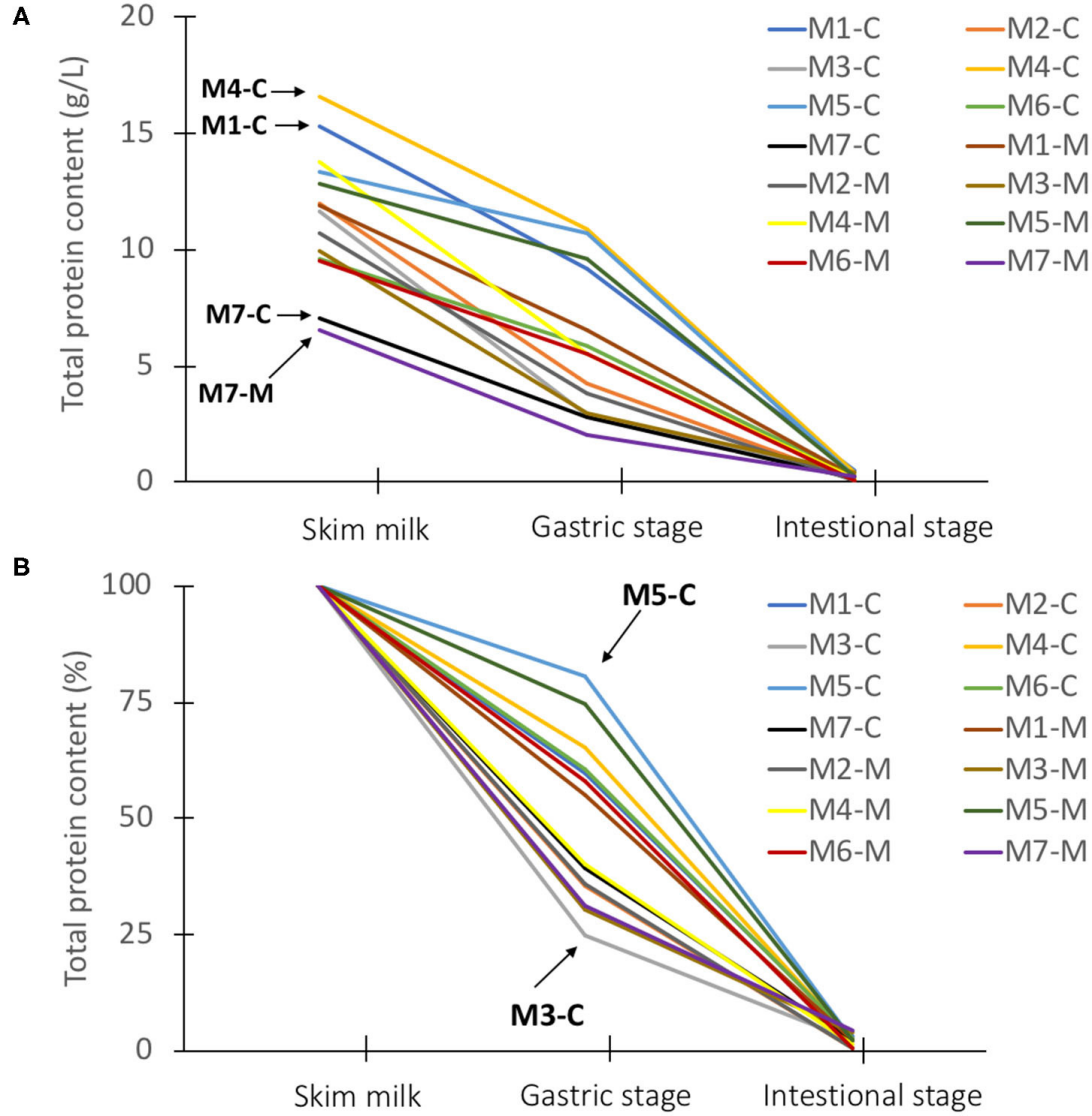
The total BCA protein content (g/L) **(A)** and total BCA protein content (%) **(B)** in skim milk for colostrum (week 1) and mature milk (week 4) from 7 Chinese mothers after dilution of the samples, and after gastric and intestinal stage. The total BCA protein concentrations (g/L) in diluted skim milk for colostrum and mature milk of the individual mothers was set to 100%. MX; X = mother. The letter behind the hyphen indicates colostrum (C) and mature milk (M).

The disappearance of human milk proteins was quantified using the BCA protein assay. Information was obtained on the total protein content before and after digestion of milk from 7 mothers and from 2 lactation periods (colostrum, week 1 and mature milk, week 4). The total BCA protein concentrations in skim milk ranged from 7.1 to 16.6 g/L for colostrum, and from 6.6 to 13.8 g/L for mature milk (Figure 1A). The lowest protein concentrations in colostrum (7.1 g/L) and mature milk (6.6 g/L) were from mother 7, whereas the highest protein concentrations in colostrum (16.6 g/L) and mature milk (15.3 g/L) were found from mothers 4 and 1, respectively (Figure 1A). The average BCA protein concentrations for colostrum and mature milk were 12.2 and 10.7 g/L, respectively. Colostrum contained higher total protein concentrations compared to mature milk, although the rate of decline varied among mothers during digestion (Figure 1A).

It can be observed that the total BCA milk protein content decreased from the start to the end of digestion with a large variation in the decline between mothers in the gastric phase (25–80%) (Figure 1B). The total BCA protein concentrations in colostrum for both mother 5 and 3 started at 13.3 and 11.7 g/L (Figure 1A), although showing the lowest (19.6%) and highest (75.3%) decline during gastric infant digestion of milk proteins (Figure 1B), respectively. It was also observed that still some undigested proteins could be found after intestinal digestion, ranging from 0.5 to 4.2% of total protein content (Figure 1B). The higher starting total protein concentrations in colostrum did not seem to be associated with a higher degree of total undigested proteins, as will be discussed in more detail later.

### The Most Abundant Proteins in Human Milk as Identified by SDS-PAGE

**Figure 2:**
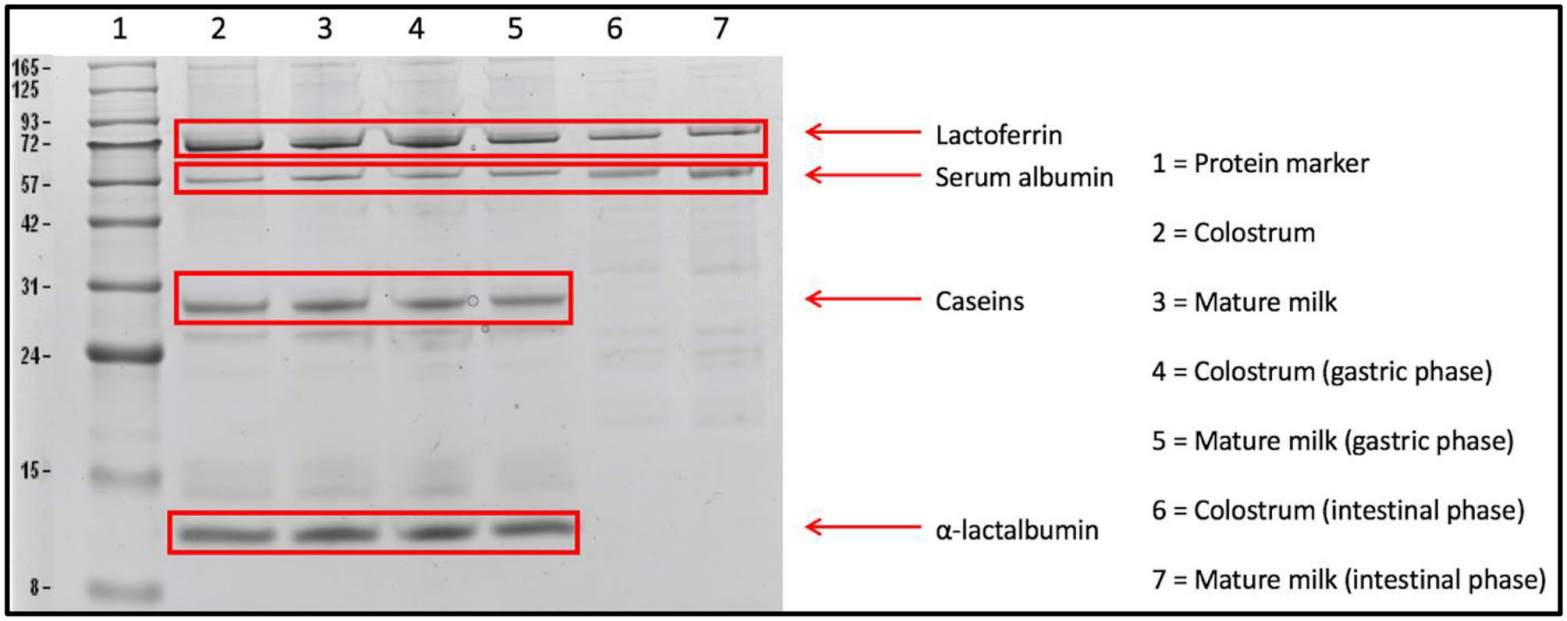
SDS-PAGE gel showing the *in vitro* digestion of colostrum (week 1) and mature milk (week 4) of one mother, highlighting lactoferrin, serum albumin, caseins, and α-lactalbumin. The total amount of protein loaded on the gels was 20 μg per sample.

The undigested proteins from human milk samples were initially monitored using an SDS-PAGE before performing in-gel digestion and the LC-MS/MS analysis. An example of an SDS-PAGE gel is given in Figure 2, showing the outcomes of *in vitro* digestion of the most abundant proteins in colostrum and mature milk from a single mother. It can be seen from Figure 2 that caseins and α-lactalbumin from colostrum and mature milk were not readily digested in the gastric phase, but were completely digested after intestinal phase. Lactoferrin and serum albumin, on the other hand, were still partially present after the intestinal phase (Figure 2). Bands of the individual human milk proteins on the SDS-PAGE gels were not quantified, however, it can be observed that proteins in mature milk were digested to a rather similar extent by the *in vitro* infant digestion model as colostrum (Figure 2). The undigested human caseins in the gastric phase, as shown in Figure 2, has not been reported before. However, this might be related to the casein micelle size in human milk, as it has been reported that the mean casein micelle size varies between mammal species (e.g., human, bovine, equine species) and that casein micelles in human milk are much smaller in size than bovine milk (24). It has also been previously reported that smaller casein micelles form a more compact and hence firmer gel network than larger casein micelles (38). The milk from bovine and human showed different degradation patterns when digested with adult gastrointestinal enzymes for 30 min at their respective pH values (pH 2.5 in gastric phase, pH 8.0 in the intestinal phase) (24). During adult gastric digestion, the caseins in human and bovine milk were poorly degraded with 69 and 39% of remaining protein, respectively (24). Further digestion of caseins with intestinal enzymes resulted in an extremely fast digestion of the caseins from all species (24), with 20% of the caseins remaining intact after 5 min, while after 30 min almost no intact caseins were left (24).

A randomized controlled trial with 12 hospitalized tube-fed preterm infants, showed that α-lactalbumin, lactoferrin, β-casein, and serum albumin were *in vivo* only partially digested in the infant's stomach (17), which matched with our findings (Figure 2). Overall, specific human milk serum proteins partially survived *in vitro* digestion, which will be further discussed while evaluating the findings obtained by LC-MS/MS.

### LC-MS/MS Evaluation of the Protein Composition Prior to Digestion

Although a more quantitative analysis of the decrease in protein content was done by the BCA protein assay, this assay only determined the total protein content (Figure 1). A general decrease of individual proteins was observed by SDS-PAGE, whereas SDS-PAGE was only able to identify some of the most abundant human milk proteins (Figure 2). LC-MS/MS provided a more complete overview of the protein composition after the *in vitro* digestion phases. Lanes of the SDS-PAGE gels were cut above the band of α-lactalbumin, which was thus used as threshold for in-gel digestion, meaning that proteinaceous material >10 KDa was assumed to be identified and quantified as intact protein.

**Figure 3:**
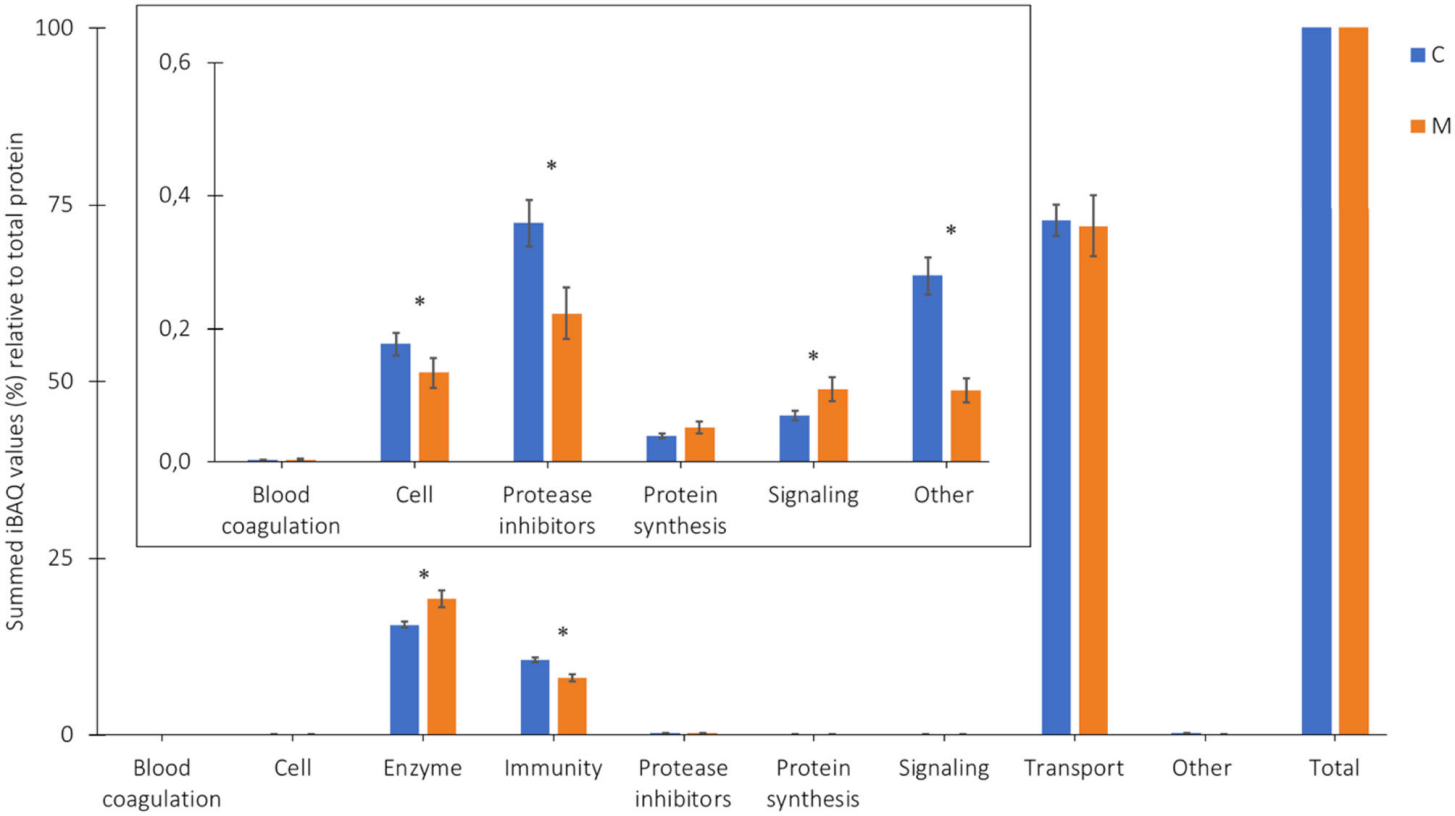
Proportion of the protein groups (%) relative to total protein, based on the summed iBAQ values obtained by LC-MS/MS, in colostrum (C, week 1) and mature milk (M, week 4) of 7 Chinese mothers averaged. ^*^Corresponding *p*-values (two-tailed *t*-test, α < 0.05). Insert illustrates zooming in on the low abundant protein groups.

The average relative protein composition in human milk from 7 Chinese mothers from 2 different lactation periods can be found in Figure 3. Proteins present in both colostrum and mature milk were dominated by immune-active proteins, enzymes, and transport proteins (Figure 3). Colostrum contained relatively higher quantities of protease inhibitors, cell proteins, and “other,” as compared to mature milk (Figure 3, insert). The relative levels of the latter protein groups were much lower than the transport, enzymes, and immune active proteins.

The higher relative levels of transport proteins in colostrum and mature milk in Figure 3, as compared to a previous study of the Chinese human serum proteome (5), might be explained by the presence of both free soluble and micellar caseins, whereas in the previous study during isolation of milk serum proteins, micellar caseins were completely removed, and only the free soluble caseins remained (5). The levels of the groups of enzymes and immune-active proteins in human milk showed a similar trend as described previously (5). The relative levels of enzymes were significantly higher in mature milk than in colostrum, whereas the relative levels of immune-active proteins were significantly higher in colostrum compared to mature milk (5).

It was also reported previously that large variation in protein composition existed among mothers, between populations and over time (5). In this study, it could also be observed that the protein composition differed among mothers and over lactation (Supplementary Figure 1). For example for Chinese mother 5, both the relative levels of immune-active proteins and enzymes increased over lactation, whereas the relative levels of transport proteins decreased from colostrum to mature milk (Supplementary Figure 1). For Chinese mother 6, immune-active proteins became relatively less abundant over lactation (Supplementary Figure 1).

### LC-MS/MS Evaluation of the Protein Composition After Gastric and Intestinal Digestion

The relative levels of undigested protein for each protein group after the gastric and intestinal digestion phase can be found in Table 1, for both colostrum and mature milk. Comparing colostrum and mature milk, higher percentages of undigested protein were found in the gastric phase for most protein groups in colostrum (Table 1). In addition, the relative levels of undigested protein for the groups protease inhibitors and protein synthesis in colostrum were the highest during gastric digestion (Table 1). For mature milk, protease inhibitors and immune-active proteins were digested to a lesser extent during gastric digestion (Table 1).

**Table 1:** Undigested human milk proteins (%) in an *in vitro* infant (0–3 months) model, based on the summed iBAQ values obtained by LC-MS/MS, categorized per biological function, for both colostrum and mature milk of 7 Chinese mothers averaged, and for gastric and intestinal digestion.

	**Colostrum (week 1)**		**Mature milk (week 4)**	
**Protein function groups**	**Gastric (%)**	**Intestinal (%)**	**Gastric (%)**	**Intestinal (%)**
Blood coagulation	25	0	7	0
Cell	31	0	18	0
Enzyme	22	0	20	0
Immunity	29	2	32	3
Protease inhibitors	44	1	35	3
Protein synthesis	41	1	14	1
Signaling	27	1	12	4
Transport	30	0	17	0
Other	22	0	22	0
**Total**	**29**	**0.3**	**18**	**0.4**

*The data has been normalized to 100% per protein group, based on pre-digestion data*.

It should be noted that a significant variation exists in protein composition from the human milk among mothers after post-intestinal treatments (Supplementary Figure 1), however, all samples had in common that the undigested levels were the highest for the immune-active proteins after the intestinal digestion phase (Supplementary Figure 1). This can also be observed in Table 1. Transport proteins were quite stable during the gastric digestion phase, but not during the intestinal digestion phase (Table 1). This might be explained by the caseins, which were the main proteins within the transport protein group. Caseins were almost completely digested after intestinal digestion, as can be observed from Table 2. The variation in protein digestion might be affected by the initial milk composition and protein profiles of the individual mothers. The static *in vitro* digestion model itself did not contribute to the variability in milk digestion amongst all 7 mothers, as the parameters of the model were similar for both lactation time points.

**Table 2:** The top 15 most abundant human milk proteins after *in vitro* infant (0–3 months) digestion, categorized per function (*N* = 7).

		**Colostrum (week 1)**		**Mature milk (week 4)**	
**Protein function**	**Name of proteins**	**Gastric (%)**	**Intestinal (%)**	**Gastric (%)**	**Intestinal (%)**
Enzymes	α-lactalbumin	22	0	20	0
	Bile salt-activated lipase	55	3	21	1
Immunity	Lactoferrin	33	1	12	3
	Ig α1-chain c-region	12	2	84	2
	Ig λ2-chain c-region	16	6	46	6
	Ig κ-chain c-region	9	8	64	12
	PIGR	39	0	61	0
	Clusterin	46	0	43	0
	Osteopontin	36	0	14	0
	β2-microglobulin	24	0	4	0
Protease inhibitor	α1-antichymotrypsin	53	1	40	1
Transport	Serum albumin	45	2	32	3
	β-casein	29	0	15	0
	α_S1_-casein	39	0	2	0
	κ-casein	37	0	23	0
Based on top 15	**Total serum proteins**	33	2	37	2
	**Total caseins**	35	0	13	0

*The data has been normalized to 100% per protein*.

### The Effect of the Higher Levels of Protease Inhibitors and Total Protein Content in Colostrum on Protein Digestion

The variation in levels of protease inhibitors and total protein were investigated in relation to the level of undigested protein, as both were hypothesized to reduce overall proteolysis. It can be observed that more intact proteins from colostrum (29%) can be found after gastric digestion compared to mature milk (18%) (Table 1). This effect is not expected to be related to the level of protease inhibitors, as no pepsin inhibitor has been found in human milk (5). Large fragments of proteins and undigested proteins leaving the stomach may be relevant *in vivo*, as they may still be biologically active before they are further degraded into smaller peptides, amino acids, and finally absorbed.

In addition, some undigested proteins could still be found after intestinal digestion (Table 1). The total protein content after intestinal digestion was similar: with 0.3 and 0.4% remaining for colostrum and mature milk, respectively (Table 1). Protease inhibitors were not completely digested during intestinal digestion, with 1 and 3% remaining for colostrum and mature milk, respectively (Table 1). In addition, it can be observed in Figure 1, that protein digestion varies between mothers, with no indications that higher total protein content in colostrum when compared to protein content in mature milk influenced the level of protein digestion. The degradation of human milk proteins during the gastric and intestinal digestion phase could not be explained by the higher levels of protease inhibitors and total protein content in colostrum than mature milk, as the r-squared values were ranging between 0 and 0.3 (Supplementary Figure 2), and as seen above (Figure 1 for total protein).

### The Survival of Individual Human Milk Proteins in an Infant *in vitro* Gastric Digestion

It can also be observed in Figure 2 that some human milk proteins remained partially intact after *in vitro* infant intestinal digestion. Table 2 is based upon the 15 most abundant proteins in Chinese human milk. The other human milk proteins still present intact after intestinal digestion can be found in Supplementary Table 1, but not in all cases for both lactation periods.

The results showed that both milk serum proteins and caseins were not completely digested during the gastric digestion phase: with 33 and 35% remaining for colostrum and 37 and 13% remaining for mature milk, respectively (Table 2). Interestingly, the levels of undigested caseins after gastric digestion were higher for colostrum than for mature milk (Table 2). The ratio between milk serum proteins and caseins in our study ranged from 33:67 to 38:62 over lactation, respectively (data not shown). The findings in Table 2 might be attributed to the hypothesis that lower levels of caseins in mature milk become easier to digest in the gastric phase than in colostrum.

The reason for the high level of gastric casein digestion (Table 2), but still partial survival of caseins, may be due to the low pepsin activity. Pepsin exerts its maximum activity at a pH of 2 (23). Another reason for this phenomenon may be the curd forming properties of casein, which happens at pH 5 (3, 17, 31). At neutral pH, caseins are negatively charged and soluble, whereas upon acidification toward their isoelectric point (4.6), as is happening under infant gastric conditions, they become less negatively charged after which caseins start aggregating into a curd. Due to the low casein level and different micelle properties in human milk, a softer curd is formed. Still, this curd is difficult to digest, which might result in a lower gastric casein digestion (11). Caseins can be degraded more easily by enzymes during intestinal digestion due to their flexible non-compact structure and lack of curd formation at pH 7 (6–8). As mentioned, many milk serum proteins survived in the gastric phase (Table 2). For example lactoferrin, which can appear in an iron-rich form, hololactoferrin, and an iron-free form, apolactoferrin. The iron-rich form of lactoferrin might stabilize the protein structure above pH 4, making hololactoferrin more resistant against enzymatic degradation in an infant *in vitro* gastric digestion. Previous studies have confirmed that lactoferrin is resistant against digestion in the infant's gastrointestinal tract (17, 24, 25), although using different *in vitro* digestion models. Another example, α-lactalbumin in colostrum and mature milk is not fully digested with 22 and 20% remaining, respectively (Table 2), which might be due to the fact that the gastric pH is too high to form the molten globule structure (3). On lowering the pH to 3 in another *in vitro* model, the acidic side chains were protonated and α-lactalbumin adopted the molten globule state, which is a less compact conformation that was easier to digest by pepsin (3). With regard to the immunoglobulins, the heavy and light chains of the different immunoglobulins are connected via disulfide bridges in human milk, making them more resistant against digestive enzymes (18), which might explain their low gastric digestion (Table 2). The reason for the large differences in rate of digestion between immunoglobulins from colostrum and mature milk remains unclear.

### The Survival of Individual Human Milk Proteins After Infant *in vitro* Intestinal Digestion

After the gastric phase, the human milk proteins where further hydrolyzed in the intestinal phase. It can be observed in Table 2 that some of the highly abundant milk serum proteins (lactoferrin, bile salt-activated lipase, immunoglobulins, α_1_-antichymotrypsin, serum albumin) from both colostrum and mature milk were still partially present after intestinal digestion (range: 1–12%) (Table 2). These specific milk serum proteins were always present in all the individual intestinal digesta samples (Supplementary Table 1). Other abundant milk serum proteins, α-lactalbumin, PIGR, clusterin, osteopontin, β_2_-microglobulin, and the 3 caseins (β-, α_S1−_, and κ-casein) from both colostrum and mature milk were almost completely digested after *in vitro* intestinal digestion (Table 2). These proteins were absent in the intestinal digesta samples of most (*N* = 6) mothers (Supplementary Table 1). Thirty-seven other human milk proteins were found after intestinal digestion (Supplementary Table 1), although, the survival levels of these proteins varied between colostrum and mature milk during digestion. Among these 37 milk serum proteins, several low abundant immunoglobulins can be found after intestinal digestion (Supplementary Table 1), but also e.g., lysozyme, α_1_-antitrypsin, and fatty acid-binding protein. Some of the milk serum proteins surviving digestion are known to be glycosylated, a feature that may have protected them against breakdown. However, as we did not analyze the level of glycosylation directly, we cannot determine whether it played a role during *in vitro* infant protein digestion.

Lactoferrin, lysozyme, immunoglobulins, antichymotrypsin, α_1_-antitrypsin, and serum albumin were previously found in the infant's feces (16), even surviving fermentation. The proportion of intact human milk proteins found in the feces varied with the age of the infants, and about 10% of the total protein intake of the breastfeed infants was undigested and appeared in the feces during the early neonatal period up to 1 month of age, while only 3% was found at 4 months of age (16). The findings in Figure 1 and Table 1 are in the same range (16). From the current study, it became clear that overall, more than 40 milk serum proteins, including several immune-active proteins (e.g., lactoferrin, immunoglobulins) and protease inhibitors (e.g., α_1_-antichymotrypsin and α_1_-antitrypsin), were still partially present intact after *in vitro* intestinal digestion. These protease inhibitors might be involved in supporting the infant's digestive tract against pathogens, as they may protect the immune-active proteins from breakdown (16, 24). Additionally, it has been reported that these protease inhibitors may affect the immune systems directly, amongst others through regulation of the complement pathway (20–22).

Undigested immunoglobulins after gastric and intestinal digestion might be important for infants in the first months of life, and might provide additional protection when the infant's immune system and digestive tract is not yet fully developed (1). This might also account for the other immune-active proteins, which survive gastric digestion, as they might still be biologically active before being further degraded during intestinal digestion. PIGR, clusterin, osteopontin, and β_2_-microglobulin are highly abundant in human milk and exert important functions for the development of the infant's immune system (5). Bile salt-activated lipase and serum albumin, which are highly abundant in human milk, were also found to be resistant (relative levels ranging from 3 to 1%) against gastrointestinal enzymes from both colostrum mature milk, and able to survive intestinal digestion in an *in vitro* infant model.

## Conclusions

This study provided, for the first time, detailed information on the digestion of proteins in an *in vitro* digestion model adapted for 0–3 months infants using both colostrum and mature milk from 7 individual Chinese mothers. LC-MS/MS was used to provide a more complete overview of the human milk protein composition before, during and after *in vitro* infant digestion. Protein digestion levels varied for the milk from the individual mothers. Large variation in total undigested protein was found between mothers after the gastric phase. Colostrum and mature milk were digested after the intestinal phase to a similar extent. In contrast to expectations, the extent of protein degradation was not directly influenced by protease inhibitors and the total protein content. Caseins were digested to a larger degree after intestinal digestion than most milk serum proteins. The relative levels of the immune-active milk serum proteins were overall the highest after intestinal digestion. The resulting intact immune-active milk serum proteins, like antibacterial proteins, might support the infant's intestine against pathogens.

## Data Availability Statement

The raw LC/MSMS data collected for this study are owned by the funder (Yili Industrial Group Company). Interested researchers can request a copy of this data by contacting the corresponding author (email: kasper.hettinga@wur.nl; tel.nr. +31-317-482401).

## Ethics Statement

The studies involving human participants were reviewed and approved by The Chinese Ethics Committee of Registering Clinical Trials (ChiECRCT-20150017). The patients/participants provided their written informed consent to participate in this study.

## Author Contributions

ME, HS, and KH conceived and planned the experiments. ME carried out the experiments and statistics and took the lead in writing the manuscript. SB contributed to sample preparation and analysis. All authors contributed to the interpretation of the results and provided critical feedback by reviewing the manuscript.

## Conflict of Interest

WW was employed by the company Inner Mongolia Yili Industrial Group. The authors declare that this study received funding from Inner Mongolia Yili Industrial Group Co. The funder had the following involvement with the study: sample collection and reviewing of the manuscript. The remaining authors declare that the research was conducted in the absence of any commercial or financial relationships that could be construed as a potential conflict of interest.

## Abbreviations

ABC: Ammonium bicarbonate
BCA: bicinchoninic acid
CaCl2: calcium chloride
DTT: dithiothreitol
FDR: false discovery rate
iBAQ: intensity based absolute quantification
IAA: iodoacetamide
LC–MS/MS: liquid chromatography–tandem mass spectrometry
LDS: lithium dodecyl sulfate
PIGR: polymeric immunoglobulin receptor
SDS-PAGE: sodium dodecyl sulfate polyacrylamide gel electrophoresis
TCA: trichloroacetic acid.
