# Supplementary material for: Degradation of Proteins From Colostrum and Mature Milk From Chinese Mothers Using an in vitro Infant Digestion Model
Source: Front Nutr. 2020 Sep 16;7:162. doi: 10.3389/fnut.2020.00162 (PMC7557360; doi:10.3389/fnut.2020.00162)
Supplement: Supplementary file 1 [file Data_Sheet_1.docx]

Supplementary Material

## Supplementary Figures and Tables

## Supplementary Figures


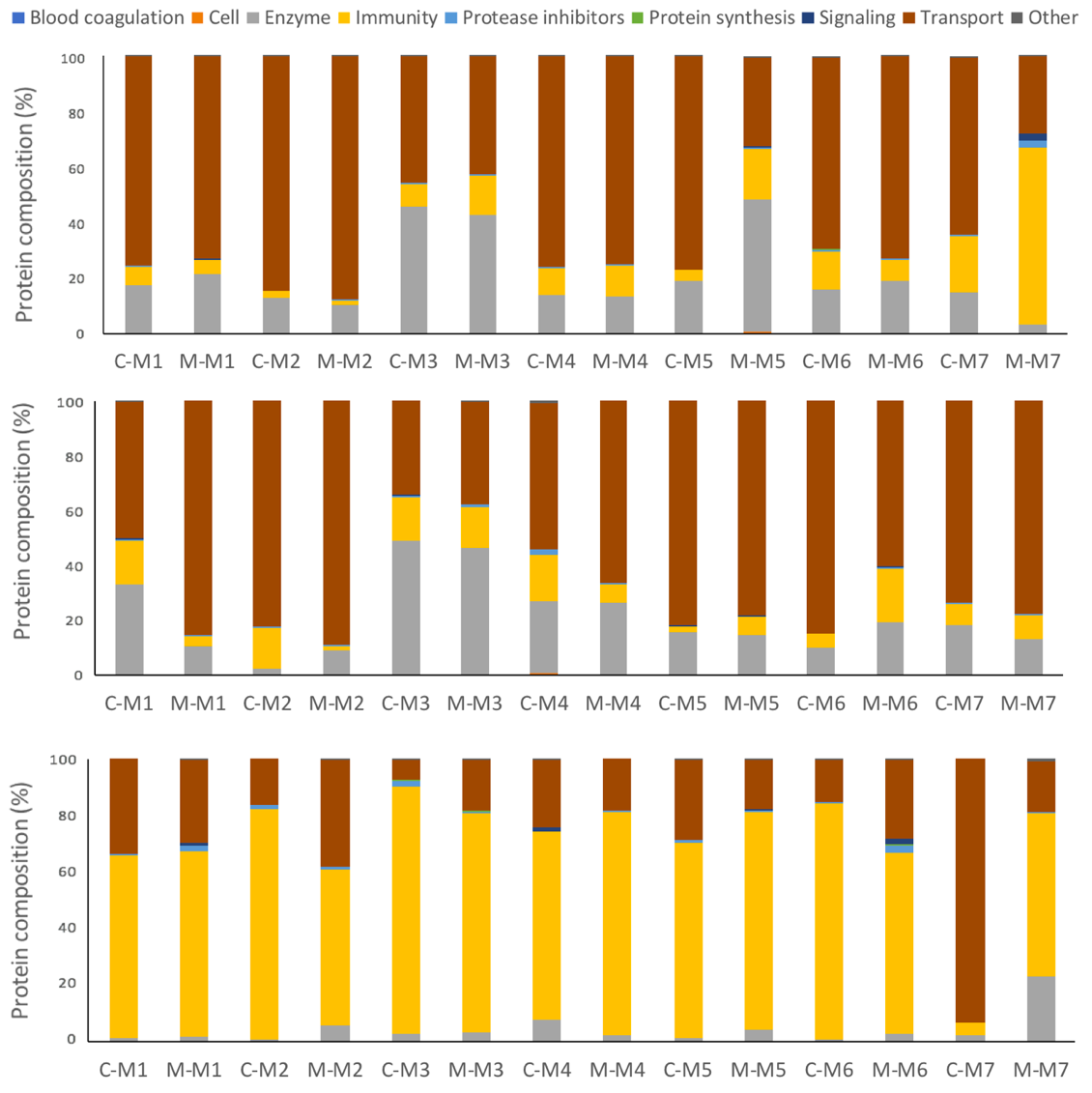


**Supplementary Figure 1.** Classes of proteins in colostrum (week 1) and mature milk (week 4) of 7 Chinese mothers **(A)**, and in an *in vitro* infant (0–3 months) digestion model for the gastric phase **(B)** and intestinal phase **(C)**, with proteins grouped having similar functions. C and M stands for colostrum and mature milk and the number behind the hyphen indicates the mother. The composition in the gastric and intestinal phase were based on total remaining proteins set on 100%.

**
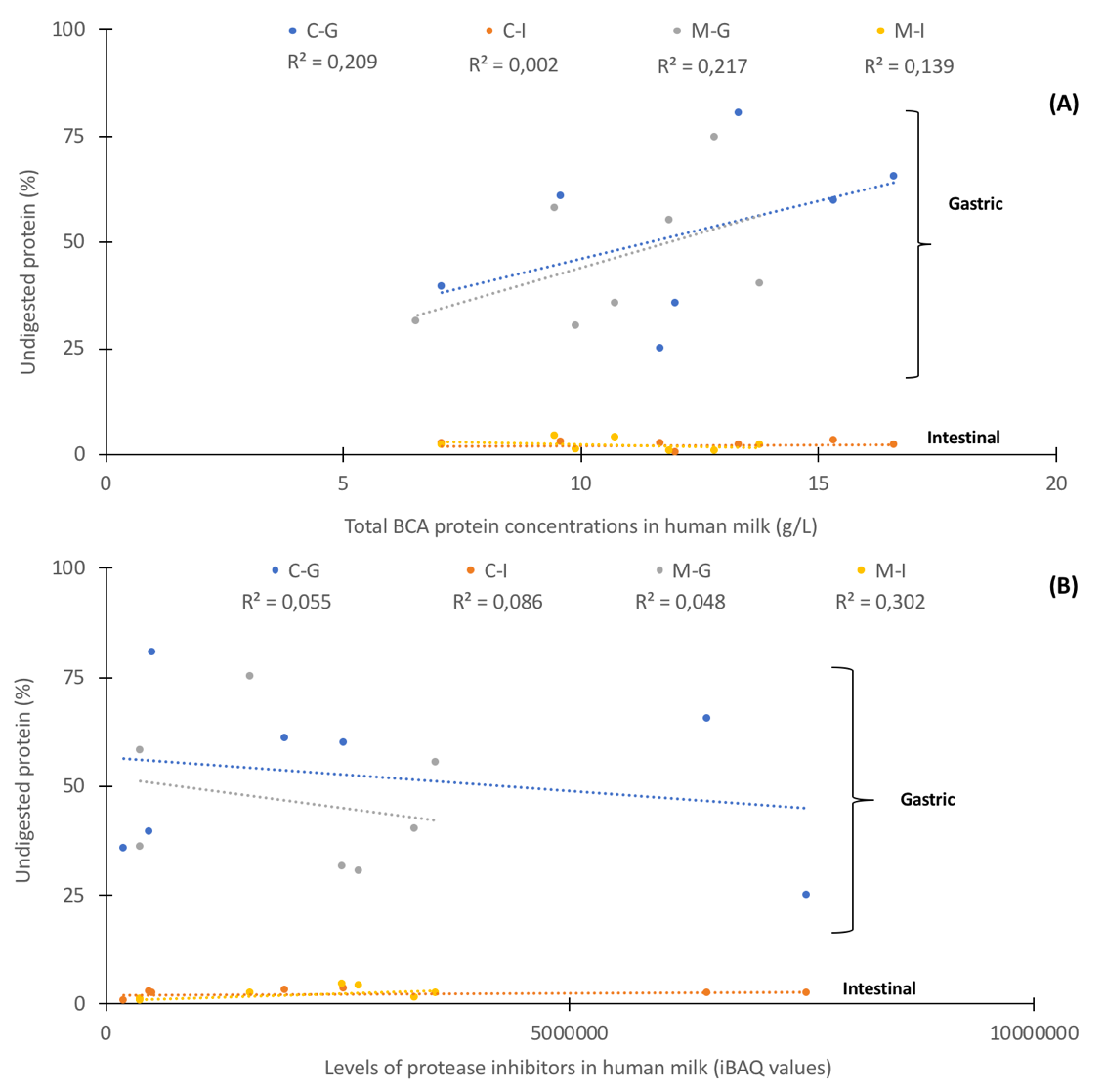
**

**Supplementary Figure 2.** The initial total protein content (based on the BCA protein assay) **(A)** and levels of protease inhibitors (iBAQ values) **(B)** in milk of 7 Chinese mothers from 2 different lactational periods (week 1 and 4) plotted versus undigested protein to gastric and to intestinal digestion. A trendline with r close to zero indicates no relationship between the two variables. The different dots represent the different samples of the mothers per lactation stage.
